# A New Generation of Brain-Computer Interfaces Driven by Discovery of Latent EEG-fMRI Linkages Using Tensor Decomposition

**DOI:** 10.3389/fnins.2017.00246

**Published:** 2017-06-07

**Authors:** Gopikrishna Deshpande, D. Rangaprakash, Luke Oeding, Andrzej Cichocki, Xiaoping P. Hu

**Affiliations:** ^1^AU MRI Research Center, Department of Electrical and Computer Engineering, Auburn UniversityAuburn, AL, USA; ^2^Department of Psychology, Auburn UniversityAuburn, AL, USA; ^3^Alabama Advanced Imaging Consortium, Auburn University and University of Alabama at BirminghamBirmingham, AL, USA; ^4^Department of Psychiatry and Biobehavioral Sciences, University of California, Los AngelesLos Angeles, CA, USA; ^5^Department of Mathematics and Statistics, Auburn UniversityAuburn, AL, USA; ^6^Skolkovo Institute of Science and Technology (Skoltech)Moscow, Russia; ^7^Nicolaus Copernicus University (UMK)Torun, Poland; ^8^Systems Research Institute, Polish Academy of ScienceWarsaw, Poland; ^9^Department of Bioengineering, University of California, RiversideRiverside, CA, USA

**Keywords:** brain-computer interface, EEG, functional MRI, simultaneous EEG/fMRI, tensor decomposition

## Abstract

A Brain-Computer Interface (BCI) is a setup permitting the control of external devices by decoding brain activity. Electroencephalography (EEG) has been extensively used for decoding brain activity since it is non-invasive, cheap, portable, and has high temporal resolution to allow real-time operation. Due to its poor spatial specificity, BCIs based on EEG can require extensive training and multiple trials to decode brain activity (consequently slowing down the operation of the BCI). On the other hand, BCIs based on functional magnetic resonance imaging (fMRI) are more accurate owing to its superior spatial resolution and sensitivity to underlying neuronal processes which are functionally localized. However, due to its relatively low temporal resolution, high cost, and lack of portability, fMRI is unlikely to be used for routine BCI. We propose a new approach for transferring the capabilities of fMRI to EEG, which includes simultaneous EEG/fMRI sessions for finding a mapping from EEG to fMRI, followed by a BCI run from only EEG data, but driven by fMRI-like features obtained from the mapping identified previously. Our novel data-driven method is likely to discover latent linkages between electrical and hemodynamic signatures of neural activity hitherto unexplored using model-driven methods, and is likely to serve as a template for a novel multi-modal strategy wherein cross-modal EEG-fMRI interactions are exploited for the operation of a unimodal EEG system, leading to a new generation of EEG-based BCIs.

## Introduction

Brain-computer interface (BCI) refers to the setup permitting the control of computers or external devices by decoding brain activity. Among the technologies being employed for decoding brain activity used in BCI, electroencephalography (EEG) presents a distinct advantage because it is non-invasive, has superior temporal resolution to allow for real-time interaction and, most importantly, is portable, widely available and practical (Mason et al., 2007). However, scalp EEG suffers from poor spatial specificity; and volume conduction through the head makes the EEG signals in different channels correlated, reducing their ability to distinguish underlying neurological processes. Consequently, the performance of EEG based BCI may be suboptimal in tasks involving deep brain structures or multiple brain structures that cannot be well-resolved with scalp EEG. With recent technical advances in neuroimaging, real-time functional magnetic resonance imaging (fMRI) has also been used for BCI. Owing to its high spatial resolution, fMRI is potentially more accurate for BCI (Weiskopf et al., 2004). However, due to its relatively low temporal resolution, high cost, restrictive environment and lack of portability, fMRI is unlikely to be used for routine BCI.

Design strategies of EEG-BCI could involve modeling the brain activity as either a dependent or an independent variable. The former case is an open loop system wherein stimulus-dependent brain activities are measured, interpreted, and used to control an external device (e.g., the P300-based speller Serby et al., 2005). This strategy can be implemented with relatively little training, but may require multiple trials to decode the brain's intention accurately, thus limiting the speed at which the external device can be operated (Blankertz et al., 2004). In the latter case, volitional control of brain activity is achieved through continuous biofeedback and the measured neural output is used to derive the signal that controls the external device. One representative approach in the latter category relies on the slow cortical potential (for example, used in cursor control) (Hinterberger et al., 2004b). Limitations of this type of approach are that they require extensive training, and their efficacy is highly variable among different individuals. Given these limitations of EEG-BCI, we hypothesize that the latent linkages between EEG and fMRI can be exploited to estimate fMRI-like features from EEG data, and hence drive an EEG-BCI using those estimated fMRI-like features. This could then allow an independently operated EEG-BCI to decode brain states in real-time. Estimation of fMRI-like features from EEG is feasible and has been tried before (De Martino et al., 2011; Meir-Hasson et al., 2014). However, the estimation accuracy has not been satisfactory when raw data has been used. Therefore, we propose the “EEG to fMRI” mapping using multi-linear subspace regression on latent variables, derived using orthogonal tensor decomposition based on the Tucker model, from both modalities. Prediction of a modality with superior spatial resolution (fMRI) from a modality with lower spatial resolution (EEG) may seem counter-intuitive; however, it is noteworthy that the missing information is provided by the transformation found using simultaneous EEG/fMRI data. Recently, Kenyan et al. (George, 2016; Keynan et al., 2016) used simultaneous EEG/fMRI and showed that by using EEG to predict fMRI signals in the amygdala, volitional control of amygdala activity by participants was achieved. Therefore, we believe that there is initial experimental basis to believe that fMRI-inspired EEG BCIs may be viable. BCI using tensor decomposition techniques has been successfully performed with EEG before (Eliseyev et al., 2011; Eliseyev and Aksenova, 2013), but not with fMRI or with a novel framework like the one we are proposing. The open loop BCI is the focus of this paper. However, the method could potentially be extended to a closed loop system with carefully designed modifications.

There has been extensive literature pertaining to the integration of EEG and fMRI, and BCIs based on each modality. We provide a brief overview here.

### Integration of EEG and fMRI

Combining EEG and fMRI data provides complementary measures of neural electrical activity at high temporal resolution and hemodynamics at high spatial resolution. For applications where brain activity is reproducible in multiple experiments (Krakow et al., 1999; Mulert et al., 2004), EEG and fMRI data can be acquired separately, though for most applications, simultaneous acquisition is desired. Recent technical advances have made simultaneous acquisition of EEG and fMRI sufficiently robust for routine applications (Salek-Haddadi et al., 2003; Herrmann and Debener, 2008; Moosmann et al., 2008; Koskinen and Vartiainen, 2009). The fundamental assumption behind any integration approach is that the signals recorded in both modalities are at least partly produced by the same neural sources. This assumption is motivated by many studies finding relationships between electromagnetic and metabolic signatures of neural activity (Logothetis et al., 2001; Mukamel et al., 2005). Specifically, it has been shown that EEG power in various frequency bands and regional blood oxygenation level dependent (BOLD) fluctuations co-vary in the resting state (Goldman et al., 2002; Mantini et al., 2007), and averaged or single trial amplitudes of event-related potential (ERP) components are correlated with BOLD fMRI signals (Nagai et al., 2004; Debener et al., 2005; Eichele et al., 2005; Hinterberger et al., 2005).

Two approaches are predominantly employed for the integration of simultaneously recorded EEG and fMRI: (1) using fMRI activations as priors for EEG source localization, and (2) examination of co-variations of the BOLD signal with different EEG signatures (Ullsperger and von Cramon, 2001; Horovitz et al., 2002, 2004; Bledowski et al., 2004; Gotman et al., 2004; Mulert et al., 2004; Nagai et al., 2004; Eichele et al., 2005; Hinterberger et al., 2005; Schicke et al., 2006). In spite of the results indicating positive correlation between some EEG signatures and the BOLD signal, it cannot be guaranteed that both measures always sample the same underlying neuronal process (Nunez and Silberstein, 2000). There may be differences in their sensitivity to different neuronal generators primarily due to the underlying differences in biophysics and a mismatch of the sampling rates (Friston et al., 1998). For instance, it has been shown that fMRI activation regions do not always provide an appropriate prior for the event-related potential (ERP) inverse problem solution (Dale et al., 2000; Dale and Halgren, 2001). These studies indicate the need for additional investigation into the linkages between latent variables underlying these modalities, since the exact relation between phase-locked and non-phase-locked ERP components with the hemodynamic response is unclear. In this paper, we propose a novel approach for discovering linkages between latent variables underlying EEG and fMRI, rather than using the acquired signals themselves.

### EEG based BCI

EEG is the method of choice for acquiring brain signals for BCI applications. An EEG based BCI system provides a communication channel between the human brain and a computer or external device based on spatio-temporal patterns extracted from EEG signals. The following problems, however, decrease the efficiency of EEG-BCI systems: poor signal to noise ratio (SNR) of EEG signatures without sufficient averaging (Lotte et al., 2007), low dimensionality of temporal EEG signatures and the fact that the acquired EEG data does not always represent the pertinent neuronal activity corresponding to the behavior which the BCI aims to decode/control. There has been some work addressing these issues. For example, it has been shown that source localization can aid the classification of task specific regions and facilitate EEG-BCI by converting the smeared scalp potential into source distribution within the brain, resulting in an improved signal (Qin et al., 2004). However, source localization is computationally intensive and is performed *post-hoc*. Hence it cannot be used to decode or control brain activity in real-time, although relatively fast algorithms for source localization have been recently reported (Becker et al., 2014). Various other feature extraction and robust classification strategies have been reported for improving the specificity and SNR of EEG features (Farwell and Donchin, 1988; Pfurtscheller et al., 1998; Penny et al., 2000; Lotte et al., 2007; Makeig et al., 2012). However, they have not been able to significantly improve the accuracy with less trials/training (Birbaumer and Cohen, 2007). This frustration was succinctly summed up by Birbaumer and Cohen: “*A large gap between the promises of invasive animal and human BCI preparations and the clinical reality characterizes the literature: while intact monkeys learn to execute more or less complex upper limb movements with spike patterns from motor brain regions alone without concomitant peripheral motor activity usually after extensive training, clinical applications in human diseases such as amyotrophic lateral sclerosis and paralysis from stroke or spinal cord lesions show only limited success*” (Birbaumer and Cohen, 2007).

### fMRI based BCI

Real-time fMRI makes it feasible to ascertain brain activity online, for decoding brain states in order to drive a BCI device (LaConte et al., 2007). Due to its spatial specificity and high dimensionality of spatial features, fMRI is very accurate for BCI applications (Sorger et al., 2012). One of the prominent approaches for decoding brain states relies on multivariate (or multiple voxel) pattern analysis (MVPA) using support vector machine (SVM) as a classifier (Norman et al., 2006), which relies on spatial patterns of voxel intensities/activations as features. Its success can be attributed to the fact that the high dimensionality of spatial patterns allows it to encode distinct signatures of underlying neural activity (Kriegeskorte et al., 2010). One major limitation of fMRI-BCI arises from the well-known time lag between neural activity and the fMRI responses detected by BOLD imaging, limiting the temporal resolution (Sitaram et al., 2007). Another severe constraint for fMRI-BCI is the restrictive MRI environment preventing it from being portable. This limitation, coupled with the substantial cost of MRI systems, makes fMRI-BCI unsuitable for routine use in practice. While simultaneous EEG/fMRI has also been used for BCI (Hinterberger et al., 2004a) and neurofeedback before (Zotev et al., 2014), the requirement that the subject be in the scanner for operating the BCI makes it impractical.

Given the complementary strengths of EEG and fMRI based BCIs, we propose experiments using an open loop P300-based speller paradigm wherein brain activity can be decoded using latent features extracted from simultaneously acquired EEG/fMRI data. Letter decoding accuracy using fMRI data is expected to outperform the accuracy obtained from only EEG. The significance of the approach lies in discovering the linkages between latent features of simultaneously acquired EEG and fMRI, such that optimal fMRI features providing excellent classification can be estimated from only EEG, when operating an EEG-only BCI. Essentially, the idea is to generalize the discovered EEG-to-fMRI transformation such that one can gain the portability and cost advantages of EEG-only BCI, and at the same time, have access to fMRI-like features (obtained from EEG) which provides higher accuracy with fewer trials. This would likely increase the speed and accuracy with which the EEG-only BCI operates, making it operationally more efficient for potential clinical populations. Finally, our approach could serve as a template for a novel multi-modal strategy wherein cross-modal EEG-fMRI interactions are exploited for the operation of a single-modal EEG system, leading to a new generation of EEG-based BCIs.

Here we put forward a framework for achieving the objectives laid out above and expand on these themes in the next section. The first objective is to discover latent linkages between EEG and fMRI. In order to achieve this, we propose the following steps. (i) *Obtain fMRI data with high temporal resolution*: The following methods can be employed: (a) Use multiband echo-planar imaging (M-EPI) (Feinberg et al., 2010), to achieve whole brain coverage with sampling intervals (TR) as short as 200 ms. (b) Use cubature Kalman filter based blind deconvolution of fMRI (Havlicek et al., 2011), to recover driving neuronal state variables with higher effective temporal resolution. (ii) *Identify EEG-fMRI transformation using simultaneously acquired EEG/fMRI data using a P300 speller based paradigm*: Obtain orthogonal tensor decomposition (based on the Tucker method) (Zhou and Cichocki, 2012) of both EEG and deconvolved fMRI data along the following dimensions: trials, voxels/channels, time and frequency. Subsequently, employ higher order multilinear subspace regression based higher order Partial Least Squares (HOPLS) model (Zhao et al., 2011) to predict the dependent variable (deconvolved fMRI) from the independent variable (EEG). The HOPLS model parameters such as the latent variables, core tensors and tensor loadings are likely to provide information about the latent EEG-fMRI relationships across the dimensions considered.

The second objective is the designing of a real-time EEG-based P300 speller using EEG-fMRI linkages. In order to achieve this, we propose the following steps. (i) *EEG-only BCI*: Perform an EEG-only experiment based on the P300 speller paradigm. (ii) *Obtain fMRI-like features*: Predict fMRI at each voxel using the transformations obtained in Objective-1 for use as input to a multivariate pattern analysis (MVPA) algorithm for decoding brain activity (the AFNI software's Cox, 1996 MVPA tool could be used). This would enable the operation of an EEG-based P300 speller in real-time mode.

## Methods

### Human subject recruitment

We suggest that, during subject recruitment, it is preferable to collect demographic data like age, in order to detect specific patterns of results, if any, which may correlate with demographic factors. For example, age related changes in P300 are well known (Juckel et al., 2012) and hence if a strong correlation exists between BCI accuracy and age, then it could be detected. On the other hand, latent EEG-fMRI linkages (as opposed to explicit relationships between raw data) may be robust against these effects, and this aspect is open to investigation.

### Objective-1: discovery of latent linkages between EEG and fMRI

In order to bring the measured fMRI signal closer to neuronal activity, we will first describe proposed approaches for acquiring fMRI data with superior temporal resolution, and blind hemodynamic deconvolution for minimizing the smoothing effect of the hemodynamic response function (HRF). Subsequently, orthogonal tensor decomposition (Kolda, 2001) using the Tucker model will be introduced as a method for subspace decomposition of EEG and deconvolved fMRI, following which the higher order partial least squares (HOPLS) model will be proposed for discovering the relationships between the latent subspace representations of EEG and deconvolved fMRI. Finally, experimental details will be provided for the P300 speller paradigm, and the entire procedure for discovering latent EEG-fMRI linkages will be put into the context of this paradigm.

#### Faster acquisition

Multiband-EPI (M-EPI) pulse sequence is a recent technique which combines two forms of multiplexing: temporal multiplexing (*m*) utilizing simultaneous echo refocused (SIR) EPI and spatial multiplexing (*n*) with multibanded RF pulses (MB), to achieve *m*×*n* images in an EPI echo train instead of the normal single image (Feinberg et al., 2010). Using 3 × 3 acceleration, TRs can be reduced up to 200 ms with whole brain coverage. This can be done without sacrificing spatial resolution. The tradeoffs between voxel size, sampling rate and coverage are given in Table 1. M-EPI data with all the options shown in Table 1 could be acquired, so that one could determine their respective effects on finding linkages between EEG and fMRI. We predict that a smaller TR would prove to be more useful than a smaller voxel.

**Table 1:** **Voxel size-sampling-coverage tradeoffs for fMRI acquisition**.

	**TR (ms)**	**Isotropic resolution (mm)**	**Coverage**
M-EPI (functional)	200	3	Whole brain
	200	2	Partial brain
	100	3	Partial brain
	800	2	Whole brain
EPI (functional)	2,000	3	Whole brain

*Red, optimized for higher temporal resolution; Blue, optimized for higher spatial resolution. The values given are notional and exact numbers will depend on the type of scanner*.

#### Blind deconvolution of HRF

The fMRI signal can be represented as a convolution of the neuronal state variables and the HRF. Since both the neuronal variables and the voxel-specific HRFs are unknown, estimating them using only the observed fMRI data is termed as blind deconvolution. Blind hemodynamic deconvolution minimizes the spatial variability of the HRF (Handwerker et al., 2004) as well as the smoothing effect of the HRF (Havlicek et al., 2011), thus increasing the effective temporal resolution of the signal. Briefly, let *k* fMRI time series be represented as *X(t)* = [*x*_*1*_*(t) x*_*2*_*(t) … x*_*k*_*(t)*]. A dynamic state-space model can be described as follows.

(1) $$\begin{array}{r} {ñ}_{t}^{k}=\left[\begin{array}{c} n_{t}^{k}\\ u_{t}^{k}\\ \theta_{t}^{k} \end{array}\right]=\left[\begin{array}{c} f\left(n_{t-1}^{k},u_{t-1}^{k},\theta_{t-1}^{k}\right)\\ u_{t-1}^{k}\\ \theta_{t-1}^{k} \end{array}\right]+\left[\begin{array}{c} q_{t-1}^{k}\\ v_{t-1}^{k}\\ w_{t-1}^{k} \end{array}\right]\; \end{array}$$

Where *n* is the neuronal state variable, *u* is the exogenous input and θ are the parameter variables of the Balloon model (Friston et al., 2000). *f* is the function which links the current neuronal state to the previous neuronal states, exogenous inputs and parameters. The subscript *t* indicates time and the superscript *k* indicates the number of the time series in the model. *q, v*, and *w* are the zero mean Gaussian state noise vectors. The observation equation, which links the state to the observation variables, is as follows.

(2) $$\begin{array}{r} x_{k}\left(t\right)=g\left({ñ}_{t}^{k}\right)+r_{t-1}\; \end{array}$$

Where *g* is the measurement function which links the state variables to the measurement variables, and *r* is the measurement noise. The inputs to the model are *x*_*k*_*(t)* and exogenous inputs *u*, which is the experimental boxcar function. As described before, the cubature Kalman filter and smoother performs very efficient joint estimation of the underlying neuronal state variables and the parameters (Havlicek et al., 2011). Also, by using a smaller time step in the estimation, the neuronal variables can be successfully estimated at an effective temporal resolution up to 10 times smaller than the TR.

#### Orthogonal tensor decomposition using the tucker model

EEG and fMRI contain rich multidimensional information which can be probed by blind source separation (BSS), i.e., decomposition into underlying sources using constraints such as independence (Niknazar et al., 2014), or low tensor rank. The most notable use of this concept is in the application of independent component analysis (ICA) to both EEG and fMRI, separately, for obtaining latent subspace representations which characterize brain function (Calhoun et al., 2001; Beckman et al., 2005) and help separate the artifacts (Onton et al., 2006). These vector subspace methods are not only limited by the number of dimensions of data that they can incorporate, but are also inferior to tensor subspace based methods for small sample size problems (Wolf et al., 2007). Published algorithms of Tensor-ICA application to fMRI are limited to 3 dimensions (Beckmann and Smith, 2005). On the other hand, multi-way tensor decomposition of EEG data based on the Tucker model has been shown to be based on solid theoretical framework, and has been demonstrated to be superior to the existing methods of feature extraction for EEG-BCI applications (Cichocki et al., 2008, 2016; Onishi et al., 2012). Importantly, this method does not use alternating least squares iterations and hence the decomposition is extremely robust and fast with well-defined identification and uniqueness conditions (Zhou and Cichocki, 2012). We propose that the same be applied to fMRI data as well, which has never been done before, though other tensor decomposition techniques such as PARAFAC has recently been applied for EEG-fMRI fusion (Ferdowsi et al., 2015). It is noteworthy that, even though other equally good higher order decompositions exist in literature (such as CP decomposition Kolda and Bader, 2009; Cichocki et al., 2015, which have been successfully used for EEG-fMRI integration Mørup et al., 2008), our proposal of the Tucker decomposition is motivated by the fact that the speed of decomposition is the fastest using the Tucker model and hence is suitable for real-time implementation required in BCI applications.

There is evidence that subspace decompositions are very effective for finding linkages between different modalities sampling similar underlying processes. Joint and parallel ICA of fMRI and EEG data has been successfully demonstrated by finding either a common mixing matrix (joint ICA) (Edwards et al., 2012; Kyathanahally et al., 2017) or a mixing matrix for each modality with the constraint that the relationship (e.g., correlation or neurovascular coupling) between them is maximized (Wu et al., 2011). We contend that inter-modality dependence may be strong between the latent variables/loadings (also referred to as components) in comparison to between the mixing matrices. This stems from the fact that the mixing matrix models the underlying biophysics, which is different for EEG and fMRI. Even if we are to constrain the relationship between EEG and fMRI mixing matrices using a neurovascular model such as the Balloon model (Friston et al., 2000), the fact remains that EEG does not represent the neuronal variables in the Balloon model. Therefore, we propose the use of HOPLS for discovering the relationships between the latent subspace representations of EEG and fMRI.

#### Multilinear subspace regression based on higher order partial least squares

Partial least squares is an established methodology for predicting a set of dependent variables *Y* from a set of independent variables *X* (Wold et al., 2001). Its optimization objective is to maximize pairwise covariance of a set of latent variables by projecting both *X* and *Y* onto a new subspace (Hou et al., 2016). Due to known limitations of *N*-way PLS for multidimensional data (Eliseyev and Aksenova, 2016), we propose that a new tensor subspace regression model called HOPLS be employed, which was proposed recently (Zhao et al., 2011, 2013).

**Figure 1:**
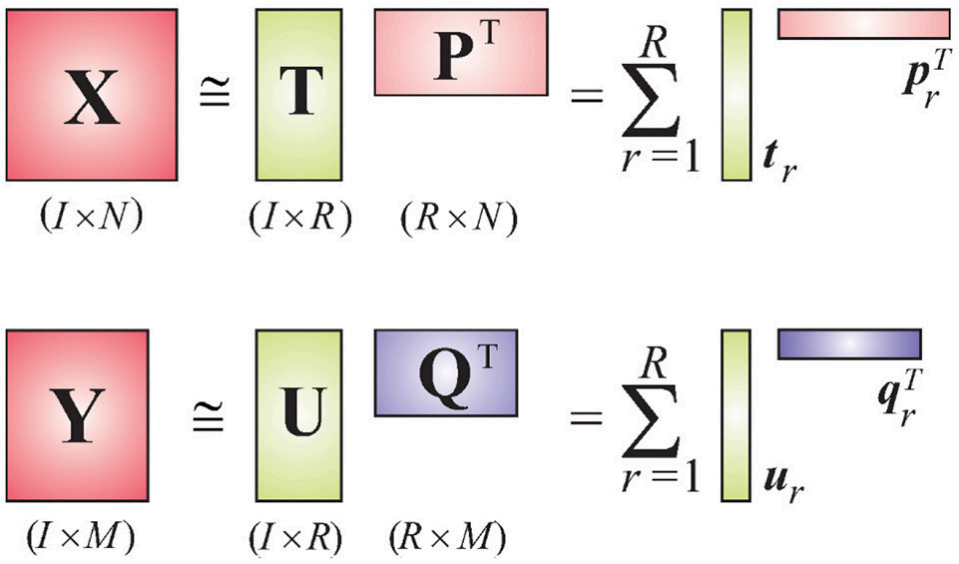
**Schematic diagram of the PLS model**.

We consider EEG data to be the independent variable *X* and deconvolved fMRI (i.e., the hidden neuronal states obtained from blind deconvolution) data to be the dependent variable *Y*. This is a reasonable assumption given the fact that the hemodynamic/metabolic activity is a secondary response to the electrical activity. The objective of the PLS method is to find a set of latent vectors that explain as much as possible the covariance between *X* and *Y*, which can be achieved by performing the following decomposition (Figure 1)

(3) $$\begin{array}{r} X=TP^{T}+E=\overset{R}{\underset{r=1}{\sum }}t_{r}p_{r}^{T}+E\\ Y=UQ^{T}+F=\overset{R}{\underset{r=1}{\sum }}u_{r}q_{r}^{T}+F, \end{array}$$

where *T* = [*t*_1_, *t*_2_, ⋯ , *t*_*R*_] and *U* = [*u*_1_, *u*_2_, ⋯ , *u*_*R*_] are matrices of *R* extracted latent variables from *X* and *Y*, respectively, and *U* will have maximum covariance with *T* column-wise. The matrices *P* and *Q* are latent vector subspace base loadings and *E* and *F* are residuals. The relation between *T* and *U* can be approximated as

(4) $$\begin{array}{r} U\approx TD, \end{array}$$

where *D* is an *R*×*R* diagonal matrix whose elements act as regression coefficients. When *X* and *Y* are tensors having the same dimensionality on the first dimension, our objective is to find their optimal subspace approximations in which their latent vectors have maximum pairwise covariance.

**Figure 2:**
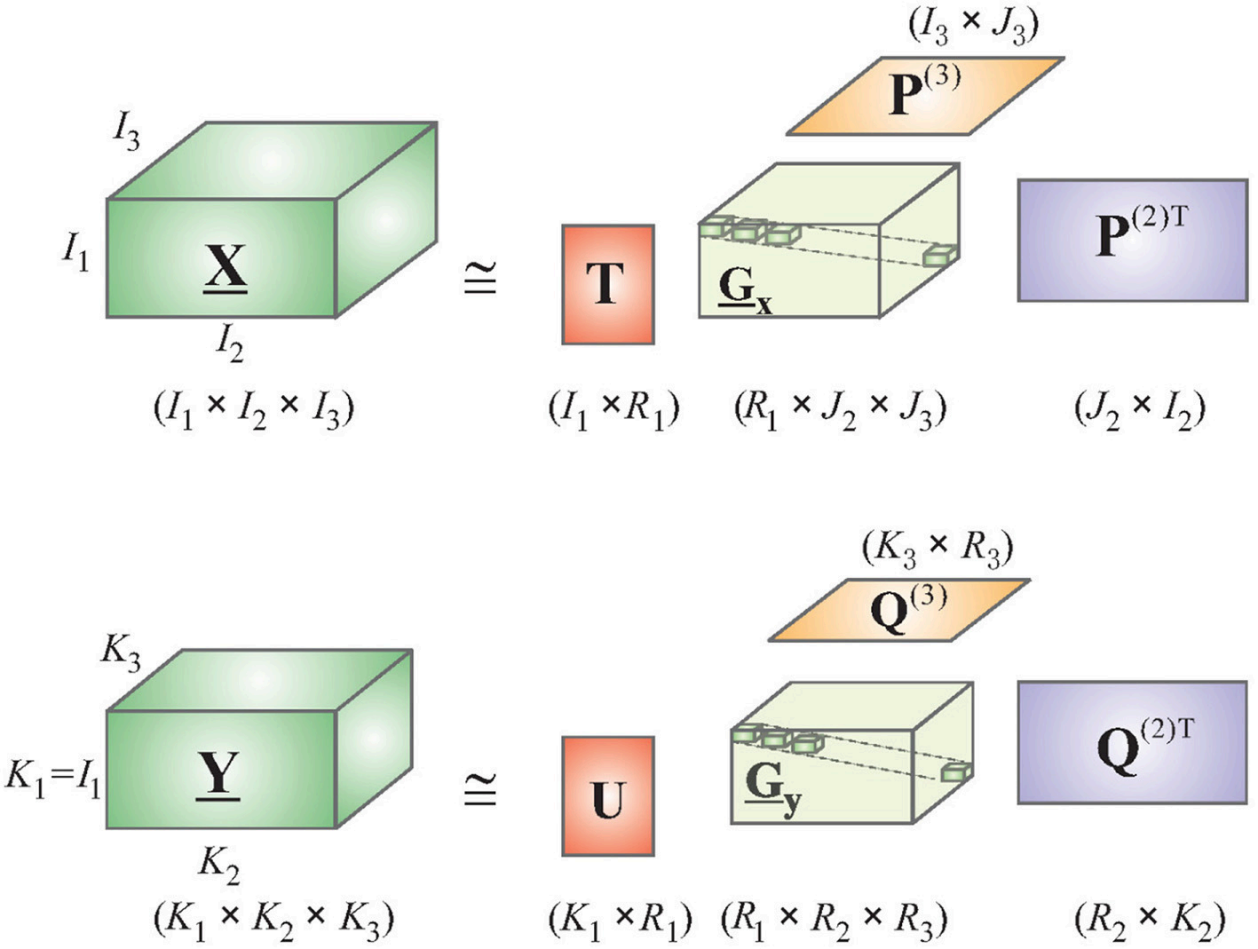
**Simplified schematic diagram of the HOPLS model**.

Consider a 4-way Tucker decomposition of EEG and deconvolved fMRI data along the following dimensions: trials, voxels/channels, time, and frequency. Here, voxels apply for fMRI and channels for EEG. A time-frequency representation of EEG and fMRI could be obtained by using the complex Morlet wavelet (Teolis, 1998). Note that the first dimension of “trials” is the same for both tensors, permitting the application of HOPLS (if performing group analysis, the number of subjects could also be used as the first dimension as it is same for EEG and fMRI). This is a very important property, which allows both EEG and fMRI to be sampled at different rates. Therefore, it is not required to downsample EEG to fMRI's temporal resolution, as done by most researchers in the EEG-fMRI comparison literature (Goldman et al., 2002; Hinterberger et al., 2005), which will lead to loss of vital temporal information. Let $\underset{-}{X}$ and $\underset{-}{Y}$ represent tensor representations of EEG and deconvolved fMRI, respectively. The new tensor subspace represented by the Tucker model can be obtained by approximating $\underset{-}{X}$ with a sum of rank- (1, *L*_2_, ⋯ , *L*_*N*_) decompositions (Figure 2), while $\underset{-}{Y}$ can be approximated by a sum of rank- (1, *K*_2_, ⋯ , *K*_*M*_) decompositions.

Using the relation in Equation (4), and integrating *D* into the core tensor, we get the HOPLS expressed as

(5) $$\begin{array}{l} \underline{X}=\underset{r=1}{\sum^{R}}\underline{G_{r}}\;\times_{1}\;t_{r}\;\times_{2}\;P_{r}^{\left(1\right)}\;\times_{3\;}\cdots \;\times_{N}\;P_{r}^{\left(N-1\right)}+\;\underline{E}\\ \underline{Y}=\underset{r=1}{\sum^{R}}\underline{D_{r}}\;\times_{1}\;t_{r}\;\times_{2}\;Q_{r}^{\left(1\right)}\;\times_{3\;}\cdots \;\times_{N}\;Q_{r}^{\left(M-1\right)}+\;\underline{F}, \end{array}$$

where *R* is the number of latent vectors, *t*_*r*_ is the *r*th latent vector, $P_{r}^{\left(n\right)}$ and $Q_{r}^{\left(m\right)}$ are loading matrices corresponding to latent vector *t*_*r*_ on mode-*n* and mode-*m*, respectively, *G*_*r*_ and *D*_*r*_ are core tensors, and ×_*r*_ is the product in the *r*th mode. Note that the core tensors model the underlying biophysics and are different for EEG and fMRI. The subspace transformation is optimized using the following objective function, yielding the common latent variable *t*_*r*_ instead of 2 latent variables as in Equation (3).

(6) $$\begin{array}{l} \;min_{\left\{P^{\left(n\right)},Q^{\left(n\right)}\right\}}||\underline{X}\;-{\left[\underline{G;}\;t,\;P^{\left(1\right)},\;\cdots ,\;P^{\left(N-1\right)}\right]}^{2}||\\ \;+||\underline{Y}-{\left[\underline{D;\;}\;t,\;Q^{\left(1\right)},\;\cdots ,\;Q^{\left(M-1\right)}\right]}^{2}||\\ \text{such}\;\text{ that}\;\left\{P^{\left(n\right)T}P^{\left(n\right)}\right\}=I_{L_{n+1}}\;and\;\left\{Q^{\left(m\right)T}Q^{\left(m\right)}\right\}=I_{K_{m+1}}\; \end{array}$$

This involves the simultaneous optimization of subspace representations and latent variable *t*_*r*_. The solution to this can be obtained by Multilinear Singular Value Decomposition (MSVD) (see Zhao et al., 2011 for more details).

#### Software for tensor decomposition

We propose that several existing toolboxes in MATLAB such as Tensor ToolBox (Bader and Kolda, 2015) and TensorLab (Sorber et al., 2015) be tested and compared for the purpose.

#### P300 speller paradigm

**Figure 3:**
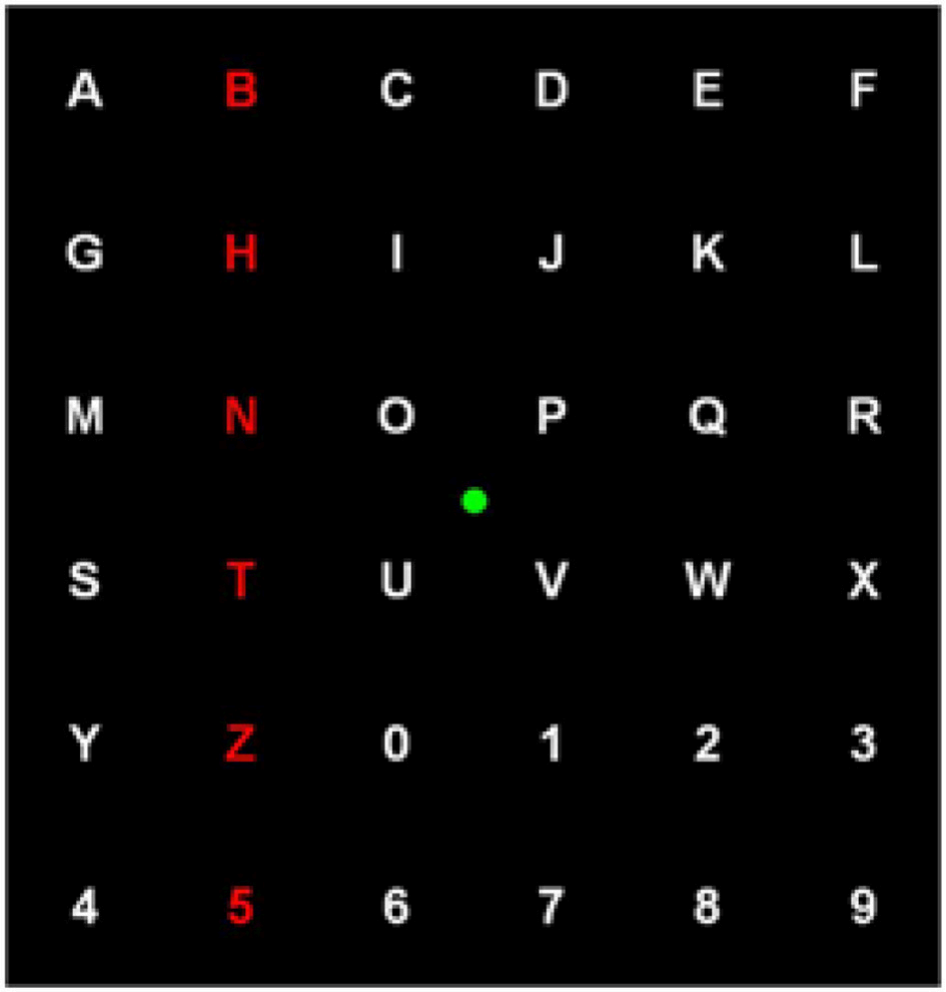
**The stimulus grid used in the P300 based speller task**.

The P300 is a positive component appearing ~300 ms after the onset of task-relevant stimuli (Hoffmann et al., 2007). To evoke the P300, subjects are asked to concentrate on a random sequence of two types of stimuli, with the target type appearing rarely in the sequence and the non-target type appearing more frequently. Whenever a target stimulus appears, a larger P300 component can be found in ERPs averaged over many trials. This phenomenon was exploited by Farwell and Donchin in the P300 speller, based on subjects' response to letters arranged in a 6 × 6 symbol matrix (Mason et al., 2007). Rows and columns are highlighted in random order, and P300 components are most strongly elicited when the row or the column containing the desired letter is flashed (Farwell and Donchin, 1988). By detecting the row and the column corresponding to the largest P300s, the letter is determined. We propose the use of a 6 × 6 stimulus grid with letters from A-Z and numbers from 0 to 9 shown in each of the cells (Figure 3). A trial in this task could be defined as a highlight (500 ms) of either a row or a column in this stimulus grid. At the beginning of each trial block, the subjects could be told a target letter/number. During subsequent trials, the subjects would need to focus eye fixation on the central green dot and see if the target letter/number is shown in the highlighted row/column. Trials could be organized in displaying cycles with each row and column being highlighted, in random order, only once in each cycle. The target/non-target trial ratio could be set to 0.2. Each subject could complete 48 trial cycles (96 target trials and 480 non-target trials) in 4 fMRI scans (12 cycles/scan) with EEG simultaneously recorded. The inter trial interval (ITI) in this task could be randomly chosen in the range of 2–5 s. The P300 may be blurred by the EOG and the EMG, and its latency can vary from 250 to 400 ms (Katayama and Polich, 2001; Spencer et al., 2001). Hence, multiple trials are often needed to detect this latency, making the speller very slow. Using our proposed method, one could aim to decrease the number of trial averages needed for high accuracy.

#### Discovering latent EEG-fMRI linkages

**Figure 4:**
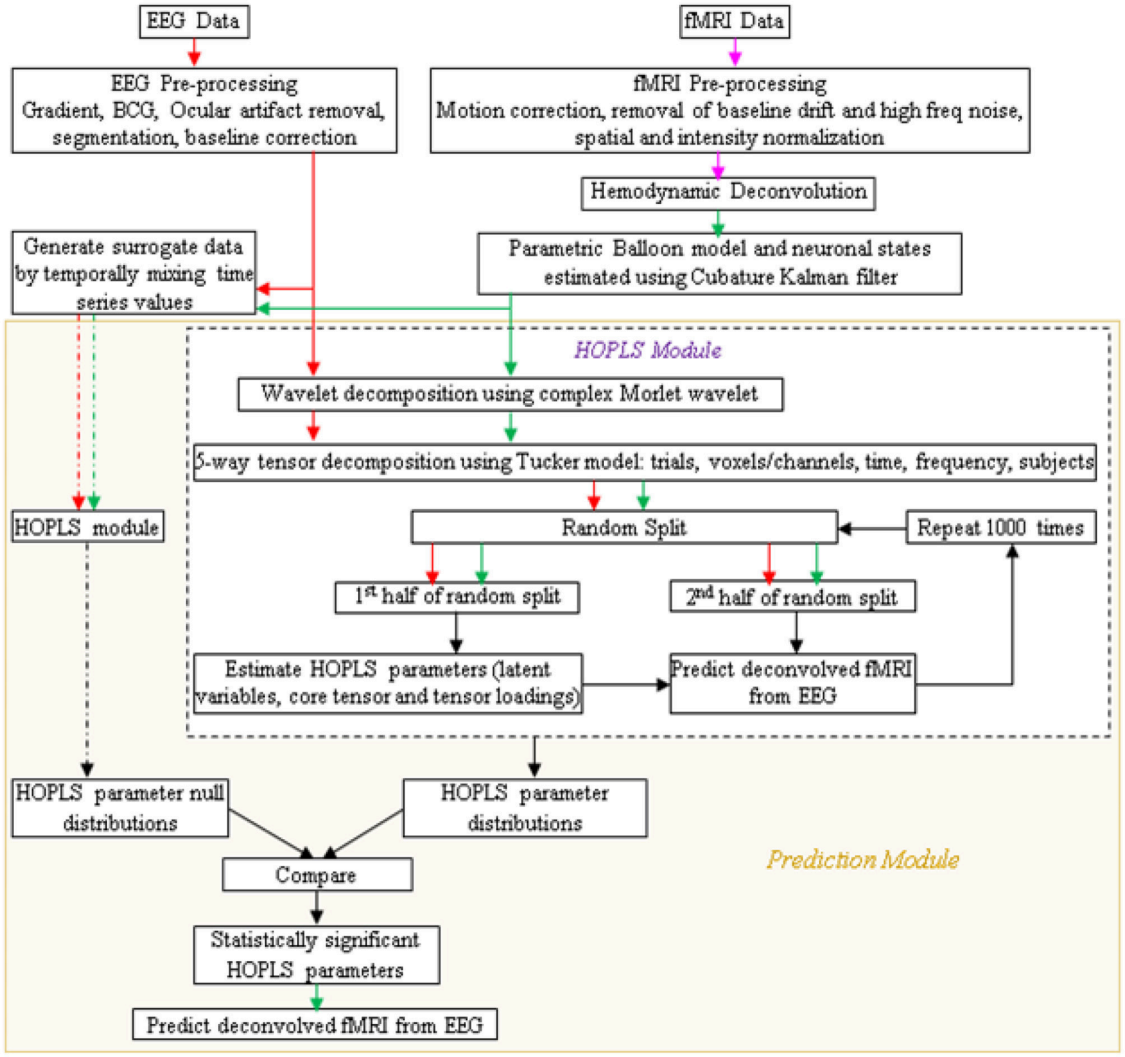
**Schematic showing the prediction of fMRI from EEG**. Arrow legend—red, EEG; magenta, fMRI; green, deconvolved fMRI; black, non-specific; dash, surrogate data.

A schematic of the proposed procedure is shown in Figure 4. After simultaneous EEG and fMRI data are acquired and pre-processed, the pre-processed EEG and deconvolved fMRI data could be represented in the time-frequency domain using the complex Morlet wavelet. Its tensor decomposition could be performed and HOPLS model parameters, i.e., latent variables, core tensor and tensor loadings, could be estimated as described before. When learning the model from multiple subjects, the decomposition could be 5-way, including the subjects as a factor. The data could be split into many random halves and HOPLS model parameters could be estimated from one of the halves as a training sample, using which the deconvolved fMRI data in the testing sample could be estimated by the corresponding EEG data using the procedure described in Zhao et al. (2011), which is essentially a forward estimation (hence, a well posed problem) of the dependent variable from the independent variable using the estimated HOPLS model, by a series of tensor operations. This would yield a distribution of HOPLS model parameters corresponding to different splits. Surrogate EEG and fMRI data could be created 10,000 times by temporally mixing the time series values in a random fashion such that the predictability between EEG and fMRI is destroyed, and the HOPLS analysis could be repeated with surrogate data. This would yield a null distribution of HOPLS model parameters corresponding to different splits and surrogates. The distribution of HOPLS model parameters obtained from original EEG and fMRI data could then be compared with the null distribution obtained from the surrogate data such that statistically significant HOPLS model parameters involved in EEG-fMRI linkage are established. This would lead to the discovery of time-frequency signatures of EEG linked to that of deconvolved fMRI.

There are several novel aspects of the proposed approach over the existing ones. First, the relevant signatures are latent and obtained without downsampling EEG to match fMRI's temporal resolution. Second, the tensor framework allows simultaneous investigation of linkages in all dimensions. Third, since deconvolved fMRI reflects neuronal states, it truly represents the linkage between electrical and metabolic neuronal states without interference from hemodynamics. Finally, the temporal correspondence between EEG and fMRI can be investigated using fMRI data with very high effective temporal resolution (≤200 ms).

### Objective-2: a real-time EEG-based P300 speller using EEG-fMRI linkages

#### BCI design

**Figure 5:**
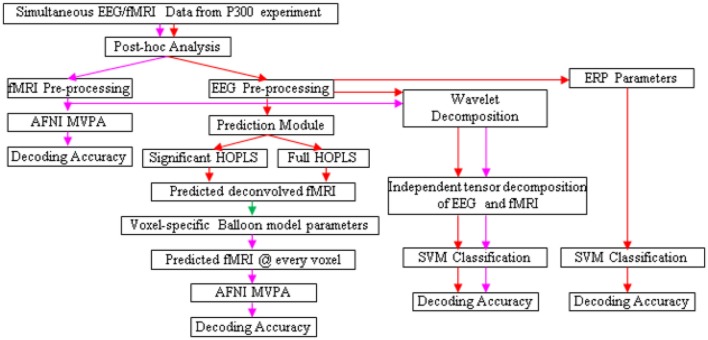
**Schematic for letter decoding from ***post-hoc*** analysis of simultaneous EEG/fMRI data**. Arrow legend—red, EEG; magenta, fMRI; green, deconvolved fMRI.

**Figure 6:**
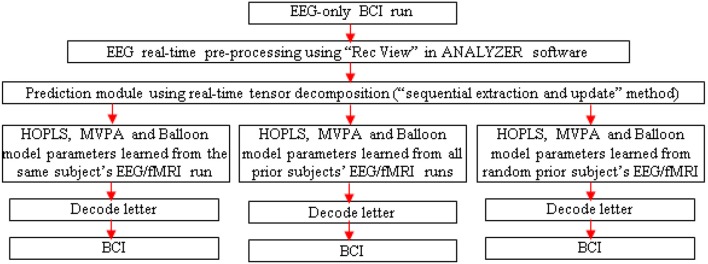
**Schematic for letter decoding from real-time analysis of EEG-only BCI run**. Arrow legend—red, EEG.

The design of the entire BCI is illustrated in Figures 5, 6. Following the acquisition of simultaneous EEG/fMRI data using the P300 speller paradigm, *post-hoc* decoding of the encoded letter could be performed from multiple features (described below) on a single trial-block basis, which is required for near real-time operation. Linear support vector machines (SVM) could be used for decoding, with standard cross-validation, complete separation of training and testing datasets (LaConte et al., 2007) and in-built feature selection algorithms such as recursive cluster elimination (RCE) (Deshpande et al., 2010). First, the encoded letter from pre-processed (but not deconvolved) fMRI data could be decoded using MVPA (which also uses SVM coupled with in-built feature selection). This accuracy is expected to be reasonably high, given the fact that fMRI spatial patterns for the oddball task are robust, specific, localized and discriminatory (Bledowski et al., 2004). Second, deconvolved fMRI data at every voxel could be predicted by EEG, as described in the previous paragraph, using statistically significant HOPLS model parameters (i.e., a sparse HOPLS model), with the non-significant ones set to zero. It could then be convolved with voxel specific HRF previously estimated using the Balloon model, resulting in estimated fMRI-like data, which could be used in an MVPA (as above) for classification.

For comparison, classification could also be performed using fMRI data estimated by a full HOPLS model (i.e., without the non-significant parameters set to zero), features obtained from subspace representations of EEG and fMRI, and standard EEG parameters such as ERP amplitude and latency. We expect that MVPA of estimated and original fMRI data would be the best performers in terms of classification accuracy.

A separate EEG-only BCI run could be performed outside the scanner, with real-time MVPA of estimated fMRI, to determine its accuracy. Since the tensor decomposition has to be real-time for the BCI to work, one could adopt the “sequential extraction and update” version of the tensor decomposition (Zhou and Cichocki, 2012).

#### Testing generalizability

Three runs could be performed with the EEG-only BCI, driven by HOPLS, MVPA and Balloon model parameters learned with the EEG/fMRI run of the same subject, all prior subjects and a random prior subject (Figure 6). These runs would aid in determining the generalizability of the proposed method across different subjects. In addition, *post-hoc* analysis of the data obtained from the EEG-only BCI could be performed involving K-fold cross validation and leave-one-out cross validation in order to determine whether the EEG to fMRI transformations learned from a set of subjects can be generalized to another set of subject/s. Further, one could investigate whether generalization (in terms of decoding accuracy) is better when the transformations learned from a given subject is applied to another subject with the same gender and similar age compared to when used with a subject of opposite gender and greater age difference. This investigation could be performed by finding a regression model between decoding accuracy and different gender/age pairings used for building the transformations using simultaneous EEG/fMRI and applying the transformations in an EEG-only BCI.

## Discussion

We proposed a novel approach for transferring the capabilities of fMRI to EEG wherein cross-modal EEG-fMRI interactions are exploited for the operation of a unimodal EEG system. Here we discuss alternative suggestions to the methodologies described earlier.

### Alternative tensor decompositions

Tucker decomposition for tensors is the higher dimensional analog of block diagonalization in matrix analysis, and produces a factorization of the tensor into a tensor with a small core, together with one change of basis matrix per mode of the tensor. PARAFAC is a refined Tucker decomposition, where the core tensor is concentrated on the super-diagonal. Here an advantage is the reliance only on matrix computations, which are very fast. Limitations occur in situations of high incoherency (high concentration in a small proportion of tensor entries) and high rank.

The more general Canonical Polyadic (CP) decomposition expresses a tensor as a sum of outer products of vectors. Computing the CP decomposition is NP Hard (Hillar and Lim, 2013), so it may be unsuitable for real-time operation of a BCI, but it might represent a model which makes more sense for our data.

Orthogonal tensor decomposition (ODECO) was recently employed by Anandkumar et al. (2014) in the case of symmetric tensors for latent variable learning. In their approach one successively extracts higher moments from the data and estimates “whitening information” for higher order symmetric tensors from lower order decompositions. For symmetric tensors of low rank, the robust tensor eigenvectors (Lim, 2005; Qi, 2005; Kolda and Mayo, 2014) (stable points of the tensor power algorithm) are orthogonal, and give the rank-one factors in the symmetric CP decomposition. One could follow this approach by analogy in the non-symmetric case. One could first estimate the covariance matrix formed by forgetting the time components in the EEG/fMRI data. Then, equipped with an estimate of the subspace of frequency-location covariance, one could reduce the computation of the full 4th order tensor by restricting to this subspace. Further, one could attempt to identify a time span for each event and extract a sub-tensor, thus reducing a large tensor decomposition problem into a collection of smaller tensor decomposition problems, and allowing one to distribute the computation, increasing efficiency on multi-core machines.

Higher order singular value decomposition (HOSVD) (De Lathauwer et al., 2000) can apply to the EEG/fMRI fusion tensor. Analogous to finding left and right singular vectors of a matrix associated to each singular value, for an *m*-mode tensor one obtains an *m*-tuple of vectors associated to a singular value, such that contraction with *m*-1 of them produces the singular value times the missing vector. Unlike ODECO, HOSVD may be used when the given tensor is not symmetric and the modes have different dimensions. In addition, the singular tuples of a tensor may be computed as critical points of a gradient on a product of spheres (Friedland and Ottaviani, 2014).

One could perform each type of decomposition and choose the one that has the best performance for the experiments, given the constraints of operating a BCI in near real-time. In addition to employing the existing algorithms for tensor decomposition, we suggest the implementation of new homotopy methods for tensor decomposition as in Hauenstein et al. (2014).

### Alternative BCI paradigms

Since the P300 speller paradigm is well-established (a PubMed search on the P300 speller returned >130 papers), one could reduce the variability of the experiment because we are insisting that all but one of the components, i.e., tensor decomposition for latent EEG-fMRI linkages, consist of well-understood, and well-established procedures. For example, if one used an untested experimental BCI paradigm while obtaining simultaneous EEG/fMRI data and our latent variable discovery were to fail, then one would not know if the failure was due to the novel paradigm or the failure of the HOPLS or MSVD algorithms. That said, the shortcomings of the P300 paradigm for BCI applications need to be factored into the risks associated with the proposal and hence alternative mitigating strategies are proposed here. The first strategy involves the operation of the P300 speller in auditory (instead of visual) mode or a mixed auditory/visual mode (Vaughan et al., 2006) in order to mitigate the eye gaze dependence of the P300 speller operated in the visual mode (Brunner et al., 2010). The second strategy involves using sensorimotor rhythms instead of the P300 to decode intentions of movement by motor imagery tasks performed by the subjects (Yuan and He, 2014).

Our approach could, in principle, be applied to open loop BCI systems which employ event-related EEG paradigms. The event-related nature of the EEG paradigms makes it easier for them to be adapted to the fMRI context by redoing the timing of events. BCIs based on EEG paradigms which are not amenable to be adapted into the simultaneous EEG/fMRI environment may not benefit from our proposed approach. As mentioned before, fMRI-inspired EEG BCIs could also be designed for closed loop BCI systems, but a more thorough specification of how that can be achieved is beyond the scope of this report. Further, it is nontrivial to extend the proposed approach to BCI systems which use implicit, rather than volitional, control. Finally, BCI systems based on neural processes that rely solely on cortical activation within a depth of 4 cm can make use of functional near–infrared spectroscopy (fNIRS) (Naseer and Hong, 2015), either alone or in combination with EEG (Koo et al., 2015). The efficacy of such systems with respect to fMRI-inspired EEG BCIs proposed is an open question and must be investigated in the future.

In summary, the salient features of the proposed EEG-to-fMRI mapping are as follows: (a) Bringing fMRI closer to EEG using faster temporal sampling of fMRI and blind deconvolution of the hemodynamic response for removing its smoothing effect and obtaining underlying neuronal variables. This is essential to achieve a good mapping between EEG and fMRI. (b) Orthogonal tensor decomposition using the Tucker method: a data-driven subspace decomposition of both EEG and deconvolved fMRI is proposed to find the underlying latent variables in multiple dimensions (such as time, frequency, trials, voxels/channels). (c) Multilinear subspace regression based on higher order partial least squares (HOPLS): Given simultaneous EEG/fMRI data, the transformation required to estimate latent variables underlying deconvolved fMRI from latent variables underlying EEG data could be obtained.

The salient features of the EEG-only BCI run driven by fMRI-like features are as follows: (d) EEG-only experiment: Using the same paradigm employed in the simultaneous EEG/fMRI experiment, it is proposed to run an EEG-only experiment. (e) The forward model: Latent variables underlying EEG data are obtained and passed through the transformation estimated using the simultaneous EEG/fMRI experiment to obtain deconvolved fMRI (using Tucker method) and raw fMRI data (using the Balloon model obtained during deconvolution) in real time. (f) Decoding: During the operation of EEG-only BCI, brain activity is decoded using fMRI-like features obtained from the forward model with acquired EEG data as inputs. The efficacy of the entire procedure is proposed to be tested using a P300-based speller BCI (though alterative BCI paradigms could also be considered to mitigate risks).

On a theoretical level, the proposed data-driven method will likely discover latent linkages between electrical and hemodynamic signatures of neural activity hitherto unexplored using model-driven methods. On a practical level, this is likely to serve as a template for a novel multi-modal strategy wherein cross-modal EEG-fMRI interactions are exploited for the operation of a unimodal EEG system, leading to a new generation of EEG-based BCIs.

## Author contributions

GD, DR, LO, AC, and XH were involved in the conception of this work; GD, AC, and XH were involved in developing the methodology; GD, DR, LO, AC, and XH contributed to writing the manuscript; GD, AC, and XH provided the resources; and GD, XH supervised the entire work.

### Conflict of interest statement

The authors declare that the research was conducted in the absence of any commercial or financial relationships that could be construed as a potential conflict of interest.
